# Are sleep disorders associated with the risk of gastrointestinal cancer?—A case–control study

**DOI:** 10.1007/s00432-023-05009-1

**Published:** 2023-06-28

**Authors:** Sven Loosen, Sarah Krieg, Andreas Krieg, Catherine Leyh, Tom Luedde, Céline Vetter, Karel Kostev, Christoph Roderburg

**Affiliations:** 1 Department of Gastroenterology, Hepatology and Infectious Diseases, Medical Faculty of Heinrich Heine University Duesseldorf, University Hospital Duesseldorf, Moorenstraße 5, 40225 Duesseldorf, Germany; 2 Department of Surgery (A), Medical Faculty of Heinrich Heine University Duesseldorf, University Hospital Duesseldorf, 40225 Duesseldorf, Germany; 3 Epidemiology, IQVIA, 60549 Frankfurt, Germany

**Keywords:** Sleep, Sleep disorders, Cancer, Gastrointestinal cancer, Epidemiology, Prevention

## Abstract

**Purpose:**

Sleep disorders are among the most common health problems worldwide and are linked to a variety of physical and mental health problems. Recently, there has been increasing evidence of an association between sleep disorders and cancer risk. We aimed to investigate this association specifically for cancers of the gastrointestinal (GI) tract.

**Methods:**

Using the DA database (IQVIA), adult patients diagnosed with GI cancer between January 2010 and December 2022 were retrospectively compared to a 1:1 propensity score-matched cohort of patients without cancer. The outcome of the study was the association between sleep disorders and subsequent diagnosis of GI cancer. To determine whether sleep disorders were more common in patients with GI cancer than in patients without GI cancer, logistic regression models were used to estimate odds ratios (ORs) with 95% confidence intervals (95% CI).

**Results:**

After matching, 37,161 cases with GI cancer and 37,161 controls without cancer were available for analysis. No association with cancer was found for sleep disorders in the overall history before the index date (OR 1.04; 95% CI 0.96–1.12), but considering sleep disorders documented within 1 year before the index date showed a positive association with GI cancer overall (OR 1.20; 95% CI 1.08–1.34). Stratified analyses by cancer site revealed higher odds of sleep disorders prior to diagnosis of gastric, pancreatic, and colorectal cancer.

**Conclusion:**

Our findings suggest that sleep disorders might be indicative of short-term health outcomes, including GI cancer, suggesting a role for sleep disorder screening in the context of cancer prevention efforts.

## Introduction

Sleep disorders are one of the most common health problems in the general population and result in significant socioeconomic and healthcare costs (Lateef et al. 2011; Ohayon 2011; Grandner 2020). Of these, insomnia and obstructive sleep apnea (OSA) have an estimated prevalence of 6–20% and 9–38%, respectively, in the general population (Riemann et al. 2017; Senaratna et al. 2017). Insomnia is characterized by delayed sleep onset, impaired sleep maintenance, reduced total sleep time, and/or early morning awakening (Sutton 2021). Obstructive sleep apnea (OSA), on the other hand, is defined by episodes of complete or partial airway collapse associated with decreased oxygen saturation or arousal from sleep (Slowik et al. 2023).

Sleep is known to be important for human health (Schlack et al. 2013; Grandner 2020). Indeed, sleep disorders have been reported to be associated with a wide range of physical and mental health problems (Schlack et al. 2013; Grandner 2020). As such, sleep disorders have been discussed as an independent risk factor for myocardial infarction, heart failure and hypertension (Laugsand et al. 2011, 2014; Palagini et al. 2013; Lin et al. 2017; Li et al. 2021), as well as stroke (Bassetti 2005). Additionally, sleep disorders have been associated with weight gain and an increased risk of developing diabetes or metabolic syndrome (Patel et al. 2008; Anothaisintawee et al. 2016). A meta-analysis by Baglioni et al. found that disrupted sleep was also a risk factor for subsequent depression, with twice the risk compared with undisturbed sleep (Baglioni et al. 2011). Other studies have linked sleep disorders to the risk of infectious and inflammatory diseases and all-cause mortality (Kripke et al. 2002; Dew et al. 2003; Irwin et al. 2004).

In recent years, there has even been growing evidence of an association between sleep and cancer (Büttner-Teleagă et al. 2021; Mogavero et al. 2021). In this context, several studies have suggested an increased risk of several cancer types including breast cancer (Davis et al. 2001), liver cancer (Hu et al. 2013), prostate cancer (Fang et al. 2015), and colorectal cancer (Stevens et al. 2011; Lin et al. 2019). However, the relationship between different sleep disorders and specific cancer site is not very clear and appears to be bidirectional (Mogavero et al. 2021). Given the limited data available to date, this study aims to investigate this association specifically for gastrointestinal (GI) cancer using a large real-world cohort from Germany based on the IQVIA Disease Analyzer (DA) database.

## Methods

### Database

This study used data from German primary care practices from the DA database (IQVIA). Details of the methodology have been published previously (Rathmann et al. 2018). In brief, the DA database contains data on demographic variables, diagnoses and prescriptions obtained in general and specialized practices in Germany. Practices included in the database are selected according to the yearly statistics of the German Medical Association, which include information on physician’s age, specialty group, community size category, and German federal state. The database covers approximately 3–6% of all private practices in Germany. It has previously been shown that the panel of practices included in the DA database is representative of general and specialized practices in Germany (Rathmann et al. 2018).

### Study population

**Fig. 1:**
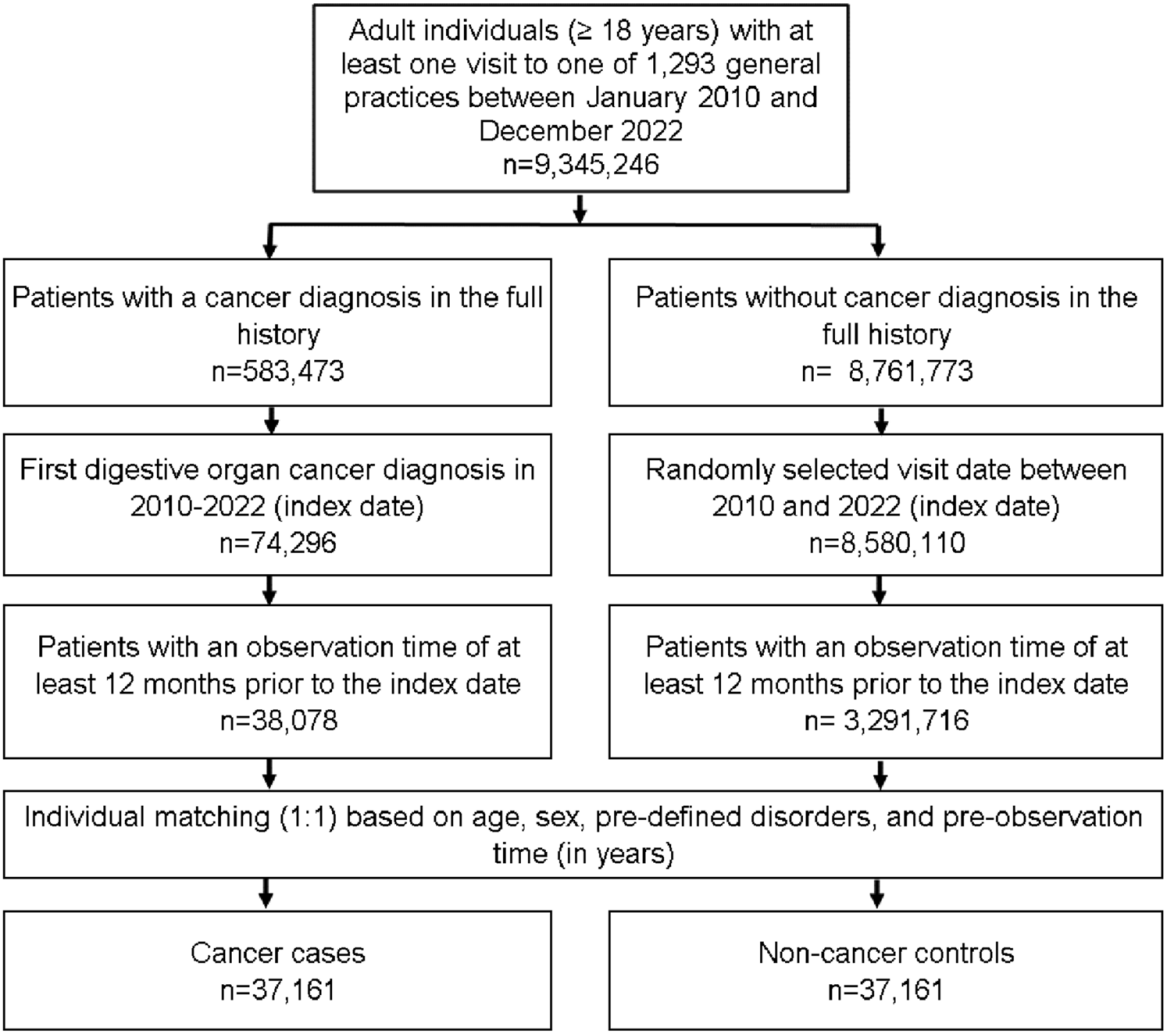
Selection of study patients

The study population included all patients aged ≥ 18 years with a cancer diagnosis of digestive tract (ICD-10 code: C15-C26) between January 2010 and December 2022 (index date) who had at least one year of observation prior to the index date. Controls were patients without cancer who were matched (1:1) by greedy propensity scores based on age, sex, their pre-diagnostic observation time in years, and pre-defined diagnoses which may be associated with increased risk of GI cancer. These diagnoses documented prior to the index date included diabetes mellitus (ICD-10: E10-E14), obesity (ICD-10: E66), chronic bronchitis and COPD (as possible proxy for smoking, ICD-10: J42-J44), diseases of esophagus, stomach, and duodenum (ICD-10: K20-K31), inflammatory bowel diseases (ICD-10: K50, K51) and diseases of liver (ICD-10: B18, K70-K77). For individuals without cancer, the index date was a randomly selected visit date between January 2010 and December 2022. The flow diagram of study participants is shown in Fig. 1.

### Study outcome

Outcome of the study was the association between sleep disorders documented prior to the index date and subsequent GI cancer diagnosis. Sleep disorders (ICD-10: G47) included insomnia, hypersomnia, circadian rhythm sleep disorders, sleep apnea, narcolepsy and cataplexy, parasomnia, sleep related movement disorders, and unspecified sleep disorders. However, general practitioner usually documented unspecified sleep disorders (ICD-10: G47.9). Other ICD-10 codes were very rarely documented. Based on the frequency of sleep disorder detail documentation, analyses were conducted for sleep disorders in total only.

### Statistical analyses

Demographic and clinical characteristics of cases and controls after 1:1 propensity-score matching were evaluated using the Wilcoxon signed-rank test for continuous variables, the McNemar test for categorical variables with two categories, and the Stuart-Maxwell test for categorical variables with more than two categories. To examine whether a history of sleep disorders was more frequent in patients with GI cancer, as compared to those with no GI cancer diagnosis, we used logistic regression models and estimated odds ratios (ORs) with 95% confidence intervals (95%CI) for sleep disorders across both patient groups. A first model analyzed the association between GI cancer and a history of sleep disorders (no matter when sleep disorder was documented in the history). In the second model, sleep disorders were classified based on the duration of disease which were calculated as number of years since sleep disorder diagnosis. Specifically, if a given sleep disorder was documented within one year prior to the GI cancer index date, time since diagnosis was set to one year. Both models were also stratified by age groups (≤ 50, 51–60, 61–70, 71–80, > 80 years), and sex. In addition to all GI cancer cases, analyses were stratified by cancer site, colorectal (ICD-10: C18-C20), gastric (ICD-10: C16), esophageal (ICD-10: C15), liver (ICD-10: C22), and pancreas cancer (ICD-10: C25). To account for multiple comparisons, we adjusted the threshold for statistical significance to p-values < 0.01. All analyses were performed using SAS 9.4 (SAS Institute, Cary, US).

## Results

### Baseline characteristics

After 1:1 matching, 37,161 GI cancer cases and 37,161 non-cancer controls were available for analyses. Mean (standard deviation, SD) age at the index date was 69.4 (12.7) years and 45.1% were female. On average, both cases and controls had 8.7 (SD 6.1) years of pre-observation time prior to index date. Case and controls had the same proportions of pre-defined diagnoses (Table 1).

**Table 1:** Characteristics of study patients after 1.1 matching

Variable	GI cancer (*n* = 37,161)	No cancer (*n* = 37,161)	*P*-value
*Age (in years)*			
Mean (SD)	69.4 (12.7)	69.4 (12.7)	1.000
≤ 50	16.5	16.5	1.000
51–60	24.3	24.3	
61–70	31.4	31.4	
71–80	31.4	31.4	
> 80	20.4	20.4	
*Sex*			
Female	45.1	45.2	0.802
Male	54.9	54.8	
Observation time prior to the index date (years), mean (SD)	8.7 (6.1)	8.7 (6.1)	0.778
Conditions documented prior to or at the index date*			
Diabetes mellitus	31.6	31.6	1.000
Obesity	12.5	12.5	1.000
Chronic bronchitis / COPD	13.9	13.9	1.000
Diseases of esophagus, gastric and duodenum	35.0	35.0	1.000
Inflammatory bowel diseases	0.9	0.8	0.259
Liver diseases	17.6	17.6	1.000

Data are absolute samples and percentages unless otherwise specified

*SD*  standard deviation

### Association between sleep disorders and cancer by cancer site

Sleep disorders in the whole history prior to the index date were not associated with cancer (OR: 1.04; 95% CI 0.96–1.12), however sleep disorders documented within one year prior to the index date was positively associated with overall GI cancer (OR: 1.20; 95% CI 1.08–1.34). Stratified analyses by cancer site showed higher odds of sleep disorders prior to gastric, pancreas, and colorectal cancer diagnosis, but these associations did not reach statistical significance (Table 2).

**Table 2:** Association between sleep disorders and subsequent GI cancer by cancer site and sleep disorder duration in patients followed in general practices in Germany

Variables / groups	Proportion (%) in patients with GI cancer	Proportion (%) in patients without cancer	OR (95% CI)	*P*-value
*Cancer in total (n = 37,161 pairs)*				
Any past sleep disorder diagnosis	4.34	4.19	1.04 (0.96–1.12)	0.326
Within ≤ 1 year	2.22	1.85	1.20 (1.08–1.34)	< 0.001
Within > 1–3 years	0.93	0.96	0.97 (0.83–1.14)	0.725
Within > 3–5 years	0.39	0.42	0.94 (0.74–1.20)	0.635
> 5 years ago	0.79	0.96	0.83 (0.70–0.98)	0.025
*Colorectal cancer (n = 17,730 pairs)*				
Any diagnosis in the history	3.93	4.00	0.98 (0.88–1.10)	0.743
≤ 1 year	2.05	1.75	1.17 (1.00–1.37)	0.047
> 1–3 years	0.83	0.89	0.93 (0.74–1.18)	0.564
> 3–5 years	0.34	0.42	0.81 (0.57–1.15)	0.232
> 5 years	0.70	0.94	0.75 (0.59–0.95)	0.016
*Gastric cancer (n = 3,566 pairs)*				
Any diagnosis in the history	4.63	4.21	1.10 (0.87–1.39)	0.409
≤ 1 year	2.27	1.60	1.42 (1.00–2.03)	0.051
> 1–3 years	0.93	1.32	0.70 (0.45–1.11)	0.134
> 3–5 years	0.36	0.41	0.90 (042–1.94)	0.781
> 5 years	1.07	0.88	1.22 (0.75–1.99)	0.434
*Esophagus cancer (n = 2,175 pairs)*				
Any diagnosis in the history	4.32	4.42	0.98 (0.72–1.32)	0.878
≤ 1 year	2.21	2.00	1.10 (0.72–1.69)	0.662
> 1–3 years	0.97	0.72	1.34 (0.68–2.64)	0.398
> 3–5 years	0.18	0.41	045 (0.13–1.49)	0.189
> 5 years	0.97	1.28	0.75 (0,42–1.35)	0.336
*Liver cancer (n = 2,271 pairs)*				
Any diagnosis in the history	5.15	4.35	1.19 (0.90–1.59)	0.219
≤ 1 year	2.51	2.47	1.02 (0.70–1.51)	0.904
> 1–3 years	1.23	0.94	1.32 (0.74–2.38)	0.348
> 3–5 years	0.44	0.20	2.24 (0.70–7.17)	0.172
> 5 years	0.97	0.74	1.32 (0.68–2.55)	0.412
*Pancreas cancer (n = 4,483 pairs)*				
Any diagnosis in the history	5.60	4.42	1.28 (1.05–1.56)	0.014
≤ 1 year	2.70	1.99	1.37 (1.03–1.83)	0.032
> 1–3 years	1.23	0.96	1.29 (0.85–1.96)	0.224
> 3–5 years	0.67	0.45	1.49 (0.83–2.69)	0.182
> 5 years	1.00	1.01	1.01 (0.66–1.54)	0.978

*OR* odds ratio, *CI* confidence interval

### Association between sleep disorders and cancer by age and sex

Overall, stratified analyses by age and sex demonstrated that positive associations between cancer and prior sleep disorder diagnoses were only observed in the age group 61–70 years (OR: 1.48; 95% CI 1.18–1.86), and in men (OR: 1.29; 95% CI 1.11–1.49) (Table 3).

**Table 3:** Association between sleep disorders and subsequent GI cancer by cancer site and sleep disorder duration in patients followed in general practices in Germany

Variables/groups	Proportion (%) in patients with GI cancer	Proportion (%) in patients without cancer	OR (95% CI)	*P*-value
*Age ≤ 50 (n=2568 pairs)*				
Any diagnosis in the history	2.69	2.05	1.32 (0.92–1.90)	0.133
≤1 year	1.75	1.24	1.43 (0.90–2.25)	0.128
>1–3 years	0.62	0.46	1.35 (0.64–2.86)	0.431
>3–5 years	0.12	0.19	0.61 (0.15–2.55)	0.497
>5 years	0.19	0.15	1.27 (0,34–4.73)	0.724
*Age 51–60 (n=5564 pairs)*				
Any diagnosis in the history	3.45	3.31	1.05 (0.85–1.28)	0.676
≤1 year	2.07	1.91	1.09 (0.83–1.42)	0.551
>1–3 years	0.74	0.78	0.94 (0.62–1.45)	0.794
>3–5 years	0.27	0.24	1.14 (0.54–2.40)	0.725
>5 years	0.38	0.38	0.99 (0.54–1.82)	0.975
*Age 61–70 (n=8119 pairs)*				
Any diagnosis in the history	4.00	3.48	1.16 (0.98–1.36)	0.079
≤1 year	2.32	1.57	1.48 (1.18–1.86)	<0.001
>1–3 years	0.79	0.95	0.84 (0.60–1.17)	0.296
>3–5 years	027	0.37	0.73 (0.42–1.26)	0.260
>5 years	0.63	0.59	1.08 (0.73–1.61)	0.710
*Age 71–80 (n=10,522 pairs)*				
Any diagnosis in the history	4.50	4.55	0.99 (0.87–1.13)	0.876
≤1 year	2.16	1.82	1.19 (0.98–1.44)	0.085
>1–3 years	0.99	0.97	1.02 (0.78–1.35)	0.873
>3–5 years	0.47	0.45	1.03 (0.69–1.53)	0.903
>5 years	0.89	1.31	0.68 (0.52–0.89)	0.004
*Age >80 (n= 6692 pairs)*				
Any diagnosis in the history	5.86	6.00	0.97 (0.84–1.12)	0.722
≤1 year	2.51	2.42	1.04 (0.83–1.29)	0.740
>1–3 years	1.30	1.30	1.00 (0.74–1.34)	0.975
>3–5 years	0.63	0.64	0.98 (0.64–1.51)	0.938
>5 years	1.42	1.65	0.86 (0.65–1.14)	0.291
*Women (n=15,023 pairs)*				
Any diagnosis in the history	4.89	4.99	0.98 (0.88–1.09)	0.696
≤1 year	2.30	2.05	1.12 (0.96–1.31)	0.160
>1–3 years	1.10	1.09	1.01 (0.81–1.25)	0.950
>3–5 years	0.47	0.50	0.94 (0.67–1.30)	0.689
>5 years	1.03	1.35	0.76 (0.62–0.94)	0.012
*Men (n=18,442 pairs)*				
Any diagnosis in the history	3.89	3.53	1.11 (0.99–1.23)	0.072
≤1 year	2.16	1.68	1.29 (1.11–1.49)	<0.001
>1–3 years	0.80	0.86	0.94 (0.75–1.17)	0.559
>3–5 years	0.33	0.35	0.95 (0.67–1.35)	0.782
>5 years	0.60	0.64	0.94 (0.72–1.22)	0.636

*OR* odds ratio, *CI* confidence interval

## Discussion

Using the IQVIA DA database, this retrospective case–control study examined the association between sleep disorders and a subsequent diagnosis of GI cancer in a real-world cohort in Germany. Adult patients diagnosed with GI cancer were compared with a control group of patients without cancer, matched 1:1 for age, sex, and diagnoses associated with an increased risk of GI cancer. Overall, there was no association between sleep disorders and cancer when the whole history before the index date was considered. Interestingly, however, when looking at the year prior to the index date, sleep disorders were positively associated with all GI cancers. These findings suggest that sleep disorders are not a risk factor for cancer, but rather an early symptom or indication of cancer. Stratified analyses by cancer site provided evidence of higher odds of sleep disorders before diagnosis of gastric, pancreatic, and colorectal cancer.

The results of our study add to an existing body of evidence linking sleep disorders to GI cancers. A retrospective case–control study by Lin et al. (Lin et al. 2019) estimated the risk of colorectal cancer in patients with sleep disorders (Lin et al. 2019) using multivariate logistic regression analysis on data from 7355 participants with colorectal cancer from the National Health Insurance Research Database of Taiwan (Lin et al. 2019). The authors found that sleep disorders were significantly associated with an increased risk of colorectal cancer. If a patient suffered from both sleep disorders and depression, the incidence of colorectal cancer was five times higher than in the control group (Lin et al. 2019).

In particular, OSA has been implicated in the development and progression of cancer; however, the evidence for this association is conflicting (Phillips et al. 2012; López et al. 2016). While some studies have found an association between OSA and cancer incidence (Lowery-Allison et al. 2018; Walker et al. 2019), other studies have failed to find this association (Mogavero et al. 2021). A retrospective nationwide analysis from Chicago examined a cohort of about 5.6 million individuals based on a health insurance database for the incidence of OSA and 12 different cancer types between 2003 and 2012. As a result, OSA was associated with only a limited number of different cancer types, including kidney cancer, pancreatic cancer, and melanoma (Gozal et al. 2016). Another study of 34,402 subjects found a differential tumor risk in OSA patients depending on the anatomic site, with a significantly higher incidence of breast, kidney, uterine, and melanoma cancers and, remarkably, a lower incidence of lung and colorectal cancers compared to the general population (Sillah et al. 2018). In contrast, a case–control study by Lee et al. examining the prevalence of colorectal neoplasia in patients with and without OSA who underwent screening colonoscopy showed that patients with OSA were approximately three times more likely to develop advanced colorectal neoplasia than controls matched for age, sex, BMI, and smoking. Consequently, the authors recommended that physicians explain the need for colonoscopy to patients with OSA (Lee et al. 2017). Additionally, a large retrospective multicenter cohort study of clinical and health administrative data from more than 30,000 individuals from Canada with suspected obstructive sleep apnea (OSA) found that OSA severity was associated with cancer incidence independent of known cancer risk factors, with the highest risk in individuals with severe hypoxemia (Kendzerska et al. 2021).

Furthermore, several studies have been published that specifically address the relationship between cancer and insomnia, but again, the results are inconsistent (Shi et al. 2020). In this context, a recent review by Shi et al. evaluated the available studies of the last 10 years after a rigorous screening and included a total of 8 cohort studies with a predominantly prospective design with 578,809 participants meeting diagnostic criteria for insomnia and 7451 cancer events (Senaratna et al. 2017). The results showed a modest 24% increased risk of cancer in people with insomnia compared with people without insomnia. Subgroup analyses revealed a significantly higher risk of cancer in studies conducted in women, but not in men (Senaratna et al. 2017). Similarly, for specific cancers, the pooled hazard ratio was significantly increased only for thyroid cancer, but not for other cancers. Despite the limited number of included studies and potential bias, the authors suggested, consistent with our conclusions, that insomnia may be an early warning sign of cancer development and may provide an opportunity for early detection and intervention (Senaratna et al. 2017).

Although the pathophysiological mechanisms underlying the association between sleep disorders and cancer are not fully understood, possible mechanisms have been discussed in the literature (Mogavero et al. 2021). It has been hypothesized that cancer may induce sleep disturbances, which in turn appear to be involved in the development and progression of cancer (Walker et al. 2019; Mogavero et al. 2021). In particular, one suggested role for the association of sleep disorders in cancer patients is the activation of an inflammatory response. Tumors produce large amounts of proinflammatory cytokines such as interleukin-1 beta (IL-1 beta), interleukin-6 (IL-6), and tumor necrosis factor-alpha (TNF-alpha), which in turn affect numerous neurotransmitters involved in sleep, including adenosine, prostaglandins, nitric oxide, and GABA (Walker et al. 2019; Mogavero et al. 2021). In this context, interestingly, sleep deprivation, short sleep duration, and other sleep disorders are associated with elevated levels of inflammatory parameters (Sillah et al. 2018). Specifically in OSA, sleep fragmentation and/or systemic intermittent hypoxia are thought to be mechanisms of carcinogenesis, inducing oxidative stress, systemic inflammation, endothelial dysfunction, and alterations in angiogenesis, sympathetic tone, immune function, and transcription factor expression that influence tumor progression (Hunyor et al. 2018; Martinez et al. 2019).

Melatonin is known to play a central role in circadian rhythm and sleep. The mechanisms of action of this hormone in cancer are not fully understood, but in addition to its role in regulating the sleep–wake cycle, melatonin is thought to be involved in inflammatory, immunological, metabolic, and neoplastic processes (Mogavero et al. 2021). Melatonin is mainly produced and secreted by the pineal gland in response to the light–dark cycle, but interestingly, it is also found in other organs such as the skin, bone marrow, and gastrointestinal tract (Acuña-Castroviejo et al. 2014). The antitumor effects of melatonin are attributed primarily to its involvement in DNA repair (Reiter 2004; Lin et al. 2019), enhancement of mitochondrial respiratory chain function, and inhibition of telomerase activity (Talib 2018). In addition, melatonin is thought to have antioxidant effects and act as a scavenger of reactive oxygen species (Talib 2018).

Sleep is hypothesized to influence the two primary effector systems, the hypothalamic-pituitary-adrenocortical axis and the sympathetic nervous system, which in turn control adaptive and innate immune responses (Irwin 2015). It has also been suggested that insufficient or disturbed sleep may decrease the release of immune-stimulating hormones such as growth hormones, prolactin, and dopamine, which could affect the natural state of the immune system (Lange et al. 2010; Lin et al. 2019).

Regarding gender differences, a review by Irwin et al. described that women appear to be more susceptible to the effects of sleep disorders on inflammatory dynamics, whereas men appear to have a higher risk of cardiovascular disease associated with sleep disorders, especially short sleep duration, and, consistent with our findings, cancer (Irwin 2015). However, future studies, especially of a prospective nature, are needed to further investigate the question of a gender-specific relationship.

In fact, only a few studies have examined the association between different types of cancer and the prevalence of sleep disorders, mainly single case reports and subjective assessments, and conducted while cancer therapy was ongoing, leading to potential bias (Mogavero et al. 2021). In particular, insomnia seems to be a very common sleep disorder in patients with malignant tumors, reported in the literature with a highly variable prevalence ranging from 19 to 63%, often associated with anxiety and depression, pain and fatigue, and surprisingly more common in men than in women (Mogavero et al. 2021). Early and adequate treatment of sleep disorders is considered important to improve the therapeutic response of cancer patients in terms of survival, quality of life, and reduction of comorbidities (Mogavero et al. 2021).

Several potential limitations of this study should be noted, which are primarily due to the study design and are unavoidable. First, the data used in the DA database (IQVIA) are based on the ICD-10 coding system for specific sleep disorders, which means that misclassification and undercoding of certain diagnoses cannot be excluded. The ICD coding for sleep disorders (ICD-10: G47) basically includes the diagnoses of insomnia, hypersomnia, circadian rhythm disorders, sleep apnea, narcolepsy and cataplexy, parasomnias, sleep-related movement disorders, and unspecified sleep disorders. It should be emphasized that primary care physicians generally coded only unspecified sleep disorders (ICD-10: G47.9), whereas specific sleep disorders were very rarely documented. Therefore, analyses were performed only for sleep disorders as a whole, and no evaluations can be made for individual subgroups of sleep disorders. Similarly, data on sleep duration, sleep onset latency, or frequency and duration of nighttime awakenings were not available. In addition, the extent of the disorders cannot be graded in our study. However, the inclusion of these parameters would be useful for follow-up studies with a different study design. It should also be considered that the ICD-10 coding of sleep disorders is mostly based on a subjective description of the patient's complaints. Furthermore, the selection of our sample may have introduced some bias, as we only used data from general practitioners in the database. Other specialties, such as gastroenterology, which are thought to have a higher detection rate for gastrointestinal cancers, were not included. Additionaly, acute or severe cancers are likely to be treated by specialists or in hospitals not included in the database. Another limitation of the study is that the DA database (IQVIA) does not include information on potential mediating effects, such as lifestyle factors like smoking, alcohol, diet, and physical activity, or information on socioeconomic status (e.g., education and income), which could potentially bias the association between sleep disorders and cancer risk. The DA database also does not provide larger panels of laboratory values or histologic parameters that would have allowed us to perform further analyses. Finally, no prognostic conclusions about cancer progression, metastasis, or survival can be drawn from our study. Thus, further studies are needed to investigate sleep problems as a modifiable risk factor associated with cancer progression. Finally, it should be stated that our study cannot establish causal relationships, only associations.

Nevertheless, it should be highlighted the large sample size as a strength of the study. Finally, to our knowledge, our study is the first population-based longitudinal study to investigate the association between sleep disorders and GI cancer in a German cohort.

## Conclusion

In conclusion, our data did not show a significant association between sleep disorders and GI cancers in the overall history. Interestingly, there was an association between sleep disorders and GI cancers when only the year of diagnosis was considered. These results suggest that sleep disorders can be interpreted as an early symptom and an indication of tumor disease. Individuals with sleep disorders may be encouraged to undergo tumor screening for early detection and treatment to positively influence disease progression and improve survival. However, large-scale prospective studies with more precise stratification of individual sleep disorders and detailed measurement of sleep duration are needed in the future to clarify the cause-effect relationship between sleep disorders and cancer.

## Data Availability

The data that support the findings of this study are available on request from the corresponding author on reasonable request.

## References

[CR1] Acuña-Castroviejo D, Escames G, Venegas C, Díaz-Casado ME, Lima-Cabello E, López LC, Rosales-Corral S, Tan DX, Reiter RJ (2014) Extrapineal melatonin: sources, regulation, and potential functions. Cell Mol Life Sci 71:2997–3025. 10.1007/s00018-014-1579-2 24554058 10.1007/s00018-014-1579-2 PMC11113552

[CR2] Anothaisintawee T, Reutrakul S, Van Cauter E, Thakkinstian A (2016) Sleep disturbances compared to traditional risk factors for diabetes development: Systematic review and meta-analysis. Sleep Med Rev 30:11–24. 10.1016/j.smrv.2015.10.002 26687279 10.1016/j.smrv.2015.10.002

[CR3] Baglioni C, Battagliese G, Feige B, Spiegelhalder K, Nissen C, Voderholzer U, Lombardo C, Riemann D (2011) Insomnia as a predictor of depression: a meta-analytic evaluation of longitudinal epidemiological studies. J Affect Disord 135:10–19. 10.1016/j.jad.2011.01.011 21300408 10.1016/j.jad.2011.01.011

[CR4] Bassetti CL (2005) Sleep and stroke. Semin Neurol 25:19–32. 10.1055/s-2005-867073 15798934 10.1055/s-2005-867073

[CR5] Büttner-Teleagă A, Kim YT, Osel T, Richter K (2021) Sleep disorders in cancer-a systematic review. Int J Environ Res Public Health. 10.3390/ijerph182111696 34770209 10.3390/ijerph182111696 PMC8583058

[CR6] Davis S, Mirick DK, Stevens RG (2001) Night shift work, light at night, and risk of breast cancer. J Natl Cancer Inst 93:1557–1562. 10.1093/jnci/93.20.1557 11604479 10.1093/jnci/93.20.1557

[CR7] Dew MA, Hoch CC, Buysse DJ, Monk TH, Begley AE, Houck PR, Hall M, Kupfer DJ, Reynolds CF 3rd (2003) Healthy older adults’ sleep predicts all-cause mortality at 4 to 19 years of follow-up. Psychosom Med 65:63–73. 10.1097/01.psy.0000039756.23250.7c 12554816 10.1097/01.psy.0000039756.23250.7c

[CR8] Fang HF, Miao NF, Chen CD, Sithole T, Chung MH (2015) Risk of cancer in patients with insomnia, parasomnia, and obstructive sleep apnea: a nationwide nested case-control study. J Cancer 6:1140–1147. 10.7150/jca.12490 26516362 10.7150/jca.12490 PMC4615350

[CR9] Gozal D, Ham SA, Mokhlesi B (2016) Sleep apnea and cancer: analysis of a nationwide population sample. Sleep 39:1493–1500. 10.5665/sleep.6004 27166241 10.5665/sleep.6004 PMC4945307

[CR10] Grandner MA (2020) Sleep, health, and society. Sleep Med Clin 15:319–340. 10.1016/j.jsmc.2020.02.017 32386705 10.1016/j.jsmc.2020.02.017

[CR11] Hu LY, Chen PM, Hu YW, Shen CC, Perng CL, Su TP, Yen SH, Tzeng CH, Chiou TJ, Yeh CM, Chen TJ, Wang WS, Liu CJ (2013) The risk of cancer among patients with sleep disturbance: a nationwide retrospective study in Taiwan. Ann Epidemiol 23:757–761. 10.1016/j.annepidem.2013.09.002 24404566 10.1016/j.annepidem.2013.09.002

[CR12] Hunyor I, Cook KM (2018) Models of intermittent hypoxia and obstructive sleep apnea: molecular pathways and their contribution to cancer. Am J Physiol Regul Integr Comp Physiol 315:R669-r687. 10.1152/ajpregu.00036.2018 29995459 10.1152/ajpregu.00036.2018

[CR13] Irwin MR (2015) Why sleep is important for health: a psychoneuroimmunology perspective. Annu Rev Psychol 66:143–172. 10.1146/annurev-psych-010213-115205 25061767 10.1146/annurev-psych-010213-115205 PMC4961463

[CR14] Irwin M, Rinetti G, Redwine L, Motivala S, Dang J, Ehlers C (2004) Nocturnal proinflammatory cytokine-associated sleep disturbances in abstinent African American alcoholics. Brain Behav Immun 18:349–360. 10.1016/j.bbi.2004.02.001 15157952 10.1016/j.bbi.2004.02.001

[CR15] Kendzerska T, Povitz M, Leung RS, Boulos MI, McIsaac DI, Murray BJ, Bryson GL, Talarico R, Hilton JF, Malhotra A, Gershon AS (2021) Obstructive sleep apnea and incident cancer: a large retrospective multicenter clinical cohort study. Cancer Epidemiol Biomarkers Prev 30:295–304. 10.1158/1055-9965.Epi-20-0975 33268490 10.1158/1055-9965.EPI-20-0975 PMC7867627

[CR16] Kripke DF, Garfinkel L, Wingard DL, Klauber MR, Marler MR (2002) Mortality associated with sleep duration and insomnia. Arch Gen Psychiatry 59:131–136. 10.1001/archpsyc.59.2.131 11825133 10.1001/archpsyc.59.2.131

[CR17] Lange T, Dimitrov S, Born J (2010) Effects of sleep and circadian rhythm on the human immune system. Ann N Y Acad Sci 1193:48–59. 10.1111/j.1749-6632.2009.05300.x 20398008 10.1111/j.1749-6632.2009.05300.x

[CR18] Lateef T, Swanson S, Cui L, Nelson K, Nakamura E, Merikangas K (2011) Headaches and sleep problems among adults in the United States: findings from the National Comorbidity Survey-Replication study. Cephalalgia 31:648–653. 10.1177/0333102410390395 21220375 10.1177/0333102410390395 PMC13112420

[CR19] Laugsand LE, Vatten LJ, Platou C, Janszky I (2011) Insomnia and the risk of acute myocardial infarction: a population study. Circulation 124:2073–2081. 10.1161/circulationaha.111.025858 22025601 10.1161/CIRCULATIONAHA.111.025858

[CR20] Laugsand LE, Strand LB, Platou C, Vatten LJ, Janszky I (2014) Insomnia and the risk of incident heart failure: a population study. Eur Heart J 35:1382–1393. 10.1093/eurheartj/eht019 23462728 10.1093/eurheartj/eht019

[CR21] Lee S, Kim BG, Kim JW, Lee KL, Koo DL, Nam H, Im JP, Kim JS, Koh SJ (2017) Obstructive sleep apnea is associated with an increased risk of colorectal neoplasia. Gastrointest Endosc 85:568-573.e561. 10.1016/j.gie.2016.07.061 27506392 10.1016/j.gie.2016.07.061

[CR22] Li L, Gan Y, Zhou X, Jiang H, Zhao Y, Tian Q, He Y, Liu Q, Mei Q, Wu C, Lu Z (2021) Insomnia and the risk of hypertension: a meta-analysis of prospective cohort studies. Sleep Med Rev 56:101403. 10.1016/j.smrv.2020.101403 33360604 10.1016/j.smrv.2020.101403

[CR23] Lin CL, Liu TC, Lin FH, Chung CH, Chien WC (2017) Association between sleep disorders and hypertension in Taiwan: a nationwide population-based retrospective cohort study. J Hum Hypertens 31:220–224. 10.1038/jhh.2016.55 27511477 10.1038/jhh.2016.55

[CR24] Lin CL, Liu TC, Wang YN, Chung CH, Chien WC (2019) The association between sleep disorders and the risk of colorectal cancer in patients: a population-based nested case-control study. In Vivo 33:573–579. 10.21873/invivo.11513. 10.21873/invivo.11513 PMC6506283 30804144

[CR25] López E, de la Torre-Luque A, Lazo A, Álvarez J, Buela-Casal G (2016) Assessment of sleep disturbances in patients with cancer: Cross-sectional study in a radiotherapy department. Eur J Oncol Nurs 20:71–76. 10.1016/j.ejon.2014.12.008 25782721 10.1016/j.ejon.2014.12.008

[CR26] Lowery-Allison AE, Passik SD, Cribbet MR, Reinsel RA, O’Sullivan B, Norton L, Kirsh KL, Kavey NB (2018) Sleep problems in breast cancer survivors 1–10 years posttreatment. Palliat Support Care 16:325–334. 10.1017/s1478951517000311 28508735 10.1017/S1478951517000311

[CR27] Martinez CA, Kerr B, Jin C, Cistulli PA, Cook KM (2019) Obstructive sleep apnea activates HIF-1 in a hypoxia dose-dependent manner in HCT116 colorectal carcinoma cells. Int J Mol Sci. 10.3390/ijms20020445 30669593 10.3390/ijms20020445 PMC6359625

[CR28] Mogavero MP, DelRosso LM, Fanfulla F, Bruni O, Ferri R (2021) Sleep disorders and cancer: State of the art and future perspectives. Sleep Med Rev 56:101409. 10.1016/j.smrv.2020.101409 33333427 10.1016/j.smrv.2020.101409

[CR29] Ohayon MM (2011) Epidemiological overview of sleep disorders in the general population. Sleep Med Res 2:1–9

[CR30] Palagini L, Bruno RM, Gemignani A, Baglioni C, Ghiadoni L, Riemann D (2013) Sleep loss and hypertension: a systematic review. Curr Pharm Des 19:2409–2419. 10.2174/1381612811319130009 23173590 10.2174/1381612811319130009

[CR31] Patel SR, Hu FB (2008) Short sleep duration and weight gain: a systematic review. Obesity (silver Spring) 16:643–653. 10.1038/oby.2007.118 18239586 10.1038/oby.2007.118 PMC2723045

[CR32] Phillips KM, Jim HS, Donovan KA, Pinder-Schenck MC, Jacobsen PB (2012) Characteristics and correlates of sleep disturbances in cancer patients. Support Care Cancer 20:357–365. 10.1007/s00520-011-1106-z 21311913 10.1007/s00520-011-1106-z

[CR33] Rathmann W, Bongaerts B, Carius HJ, Kruppert S, Kostev K (2018) Basic characteristics and representativeness of the German disease Analyzer database. Int J Clin Pharmacol Ther 56:459–466. 10.5414/cp203320 30168417

[CR34] Reiter RJ (2004) Mechanisms of cancer inhibition by melatonin. J Pineal Res 37:213–214. 10.1111/j.1600-079X.2004.00165.x 15357667 10.1111/j.1600-079X.2004.00165.x

[CR35] Riemann D, Baglioni C, Bassetti C, Bjorvatn B, Dolenc Groselj L, Ellis JG, Espie CA, Garcia-Borreguero D, Gjerstad M, Gonçalves M, Hertenstein E, Jansson-Fröjmark M, Jennum PJ, Leger D, Nissen C, Parrino L, Paunio T, Pevernagie D, Verbraecken J, Weeß HG, Wichniak A, Zavalko I, Arnardottir ES, Deleanu OC, Strazisar B, Zoetmulder M, Spiegelhalder K (2017) European guideline for the diagnosis and treatment of insomnia. J Sleep Res 26:675–700. 10.1111/jsr.12594 28875581 10.1111/jsr.12594

[CR36] Schlack R, Hapke U, Maske U, Busch M, Cohrs S (2013) Frequency and distribution of sleep problems and insomnia in the adult population in Germany: results of the German Health Interview and Examination Survey for Adults (DEGS1). Bundesgesundheitsblatt Gesundheitsforschung Gesundheitsschutz 56:740–748. 10.1007/s00103-013-1689-2 23703493 10.1007/s00103-013-1689-2

[CR37] Senaratna CV, Perret JL, Lodge CJ, Lowe AJ, Campbell BE, Matheson MC, Hamilton GS, Dharmage SC (2017) Prevalence of obstructive sleep apnea in the general population: a systematic review. Sleep Med Rev 34:70–81. 10.1016/j.smrv.2016.07.002 27568340 10.1016/j.smrv.2016.07.002

[CR38] Shi T, Min M, Sun C, Zhang Y, Liang M, Sun Y (2020) Does insomnia predict a high risk of cancer? A systematic review and meta-analysis of cohort studies. J Sleep Res 29:e12876. 10.1111/jsr.12876 31352687 10.1111/jsr.12876

[CR39] Sillah A, Watson NF, Schwartz SM, Gozal D, Phipps AI (2018) Sleep apnea and subsequent cancer incidence. Cancer Causes Control 29:987–994. 10.1007/s10552-018-1073-5 30120643 10.1007/s10552-018-1073-5 PMC6162141

[CR40] Slowik JM, Sankari A, Collen JF (2023). Obstructive Sleep Apnea. StatPearls. Treasure Island (FL), StatPearls Publishing Copyright © 2023, StatPearls Publishing LLC

[CR41] Stevens RG, Hansen J, Costa G, Haus E, Kauppinen T, Aronson KJ, Castaño-Vinyals G, Davis S, Frings-Dresen MH, Fritschi L, Kogevinas M, Kogi K, Lie JA, Lowden A, Peplonska B, Pesch B, Pukkala E, Schernhammer E, Travis RC, Vermeulen R, Zheng T, Cogliano V, Straif K (2011) Considerations of circadian impact for defining “shift work” in cancer studies: IARC Working Group Report. Occup Environ Med 68:154–162. 10.1136/oem.2009.053512 20962033 10.1136/oem.2009.053512

[CR42] Sutton EL (2021) Insomnia. Ann Intern Med 174:Itc33-itc48. 10.7326/aitc202103160. 10.7326/AITC202103160 33683929

[CR43] Talib WH (2018) Melatonin and Cancer Hallmarks. Molecules. 10.3390/molecules23030518 29495398 10.3390/molecules23030518 PMC6017729

[CR44] Walker WH, Borniger JC (2019) Molecular mechanisms of cancer-induced sleep disruption. Int J Mol Sci. 10.3390/ijms20112780 31174326 10.3390/ijms20112780 PMC6600154

